# Research on Pepper External Quality Detection Based on Transfer Learning Integrated with Convolutional Neural Network

**DOI:** 10.3390/s21165305

**Published:** 2021-08-05

**Authors:** Rui Ren, Shujuan Zhang, Haixia Sun, Tingyao Gao

**Affiliations:** College of Agricultural Engineering, Shanxi Agriculture University, Jinzhong 030801, China; ruiren@stu.sxau.edu.cn (R.R.); sunhaixia@sxau.edu.cn (H.S.); gaoty@stu.sxau.edu.cn (T.G.)

**Keywords:** pepper, image processing, quality detection, transfer learning, convolutional neural network, classification

## Abstract

A pepper quality detection and classification model based on transfer learning combined with convolutional neural network is proposed as a solution for low efficiency of manual pepper sorting at the current stage. The pepper dataset was amplified with data pre-processing methods including rotation, luminance switch, and contrast ratio switch. To improve training speed and precision, a network model was optimized with a fine-tuned VGG 16 model in this research, transfer learning was applied after parameter optimization, and comparative analysis was performed by combining ResNet50, MobileNet V2, and GoogLeNet models. It turned out that the VGG 16 model output anticipation precision was 98.14%, and the prediction loss rate was 0.0669 when the dropout was settled as 0.3, learning rate settled as 0.000001, batch normalization added, and ReLU as activation function. Comparing with other finetune models and network models, this model was of better anticipation performance, as well as faster and more stable convergence rate, which embodied the best performance. Considering the basis of transfer learning and integration with strong generalization and fitting capacity of the VGG 16 finetune model, it is feasible to apply this model to the external quality classification of pepper, thus offering technical reference for further realizing the automatic classification of pepper quality.

## 1. Introduction

Pepper is one of the most widely planted economic crops in the world, which has the highest vitamin C content in vegetables. Pepper contains rich capsaicin and has a strong spicy taste and special effect in seasoning. Moreover, pepper is also deemed as a major source for green food coloring due to the rich content of paprika oleoresin in pepper pericarp [1]. In recent years, worldwide demand for pepper has been continuously increasing. Different kinds of external defects could influence the quality of pepper. At present, the external quality classification of pepper mainly depends on manual selection, which is of low efficiency, strong subjectivity, and large work load. Such a situation, to a great extent, has become a limitation to the large-scale production and promotion of the pepper industry. Therefore, an automatic and smart technological solution for pepper external quality classification is urgently needed. This solution would be of significant production meaning and economic benefit to the development of the pepper industry.

With the development of computer technology, image processing technology based on machine learning has been applied to agricultural product defect detection. Based on computer visual technology, Dian Rong et al. adopted a sliding comparison window local segmentation algorithm for orange external defect detection and realized 97% precision [2]. Habib et al. used color feature extraction from papaya images to realize papaya disease identification by classifying disease features with support vector machine and achieved 90.15% output precision [3]. Liu Jun et al. used a radial basis support vector machine identification model for the identification of three kinds of walnut external defects including cracks, broken shells, and black spots and realized 90.21% total identification [4]. Zhao Juan et al. used a digital process for external defect analysis for apples [5]. They proposed judging the defect size of fruit with the area ratio and realized a 92.5% total precision rate.

Over recent years, domestic and overseas researchers mainly used deep learning technology for external quality detection of agriculture products. Traditional machine learning requires manual extraction of image feature information, which contains the risk of low test precision due to lack of integrity in the extracted information. It can also result in large amounts and low efficiency in calculation for datasets with a large sample size. Compared with machine learning, convolutional neural network (CNN) has a shared convolutional kernel, which results in ease when using high-dimension data processing and allows for the capability of automatic feature extraction. CNN automatically extracts different features of images with convolution operation. The convolutional layer also includes activation function to assist in the expression of complex features [6]. Researchers adopted convolutional neural network models including VGGxNet [7], ResNet [8], MobileNet [9], and GoogLeNet [10] for studies on agricultural product maturity detection [11,12,13], defect detection [14,15,16], and grading [17,18,19]. Costa proposed a deep neutral network model for detection towards external defects on tomatoes [20]. The model was based on ResNet. It used feature extraction and finetune models to detect external defects on tomatoes. Average precision of the model was 94.6%, which realized relatively precise classification of tomatoes. Momeny et al. conducted cherry identification and grading according to regularity of the fruit shape. Using the traditional CNN model with generalization capability improved, they finally realized a 99.4% classification precision rate [21]. Xue et al. used the GoogLetNet deep transferring model for detection towards defects on apples [22]. The final identification precision rate was 91.91%. This method was of good generalization capability and robustness. Lei et al. proposed a dried jujube classification method based on a double-branch deep-fusion convolution neural network (DDFNet) in which network structure was designed as a double-branch structure network [23]. The network was fused into the fusion module by squeezing and expanding feature image output by convolutional layer. This method realized 99.6%, 99.8%, 98.5%, and 99.2% precision rates on plump, skinny, cracked, and inferior jujubes, respectively. Li proposed an improved approximate VGG model for quick detection towards external defects on hami melon [24], which output the best model training performance at a 0.001 learning rate. The precision rate in the test was 97.1%. Deep learning has good applicability and strong learning ability, which provides a reference and feasibility basis for the application of convolutional neural network in agricultural product quality detection. However, reports about pepper external quality classification are very rare. In order to solve the problem of automatic classification of pepper appearance quality, it is necessary to study the appearance quality of pepper, which provides a feasible basis for the classification of pepper external quality based on deep learning.

This research adopted a transfer-learning-based VGG 16 model for model optimization with combined methods of finetuned dropout and learning rate. To promote convergence rate, stability, fitting capability, and precision of the network, the influence of adding a batch normalization layer on the model was tested; the influence of different activation functions on the performance of the model was tested; pepper selection model was established and compared with ResNet 50, MobileNet V2, and GoogLeNet models in transfer learning after parameter optimization. This research is the first to combine machine vision with deep learning used to detect the quality of pepper for the first time. The purpose of this study is to explore a low-cost, efficient, and non-destructive method to detect the quality of pepper. In order to solve the problem of low efficiency of artificial sorting quality of pepper, to realize the accurate classification and discrimination of the external quality of pepper after picking, and to provide technical support for the development of the pepper industry.

## 2. Materials and Methods

### 2.1. Materials and Equipment

The research adopted Capsicum annuum as research object. The image collection system includes a darkroom, a camera with a USB port (S-Yue SY8031, Shenzhen, China), two LED light bars, a luminous slab, and a lifting platform. Background photograph for pepper is white, to help distinguish the pepper from the background. The angle and luminance of light bars were adjusted to eliminate a shadow in pepper image as much as possible. The color temperature of LED light bars was 5500 K. Distance to the camera was 11 cm. This could avoid color deviation when the camera took pictures.

**Figure 1:**
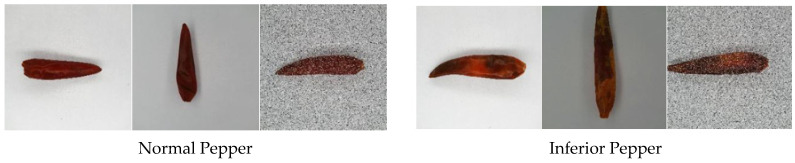
Part of the pepper dataset.

In accordance with NY/T 944-2006 standard, peppers are categorized into normal pepper and inferior pepper. A normal pepper is in regular shape, clean, and bright red color. An inferior pepper may exhibit defects including black spots, worm damage, corrugate skin, etc. The experiment collected 776 images, including 400 pictures of normal peppers and 376 pictures of inferior peppers. The research expanded the sample pepper images by operations including 90°-, 180°-, and 270° rotation, illumination changing, contrast changing, and noise adding. Part of the pepper dataset is shown in Figure 1. To satisfy the needs of neural network training and strengthen the robustness of the classification model, all images were configured to 224 × 224 before being input into the CNN. At last, 2400 pictures of normal peppers and 2256 pictures of inferior peppers were collected.

The research used Windows 10 operating system, Intel Core i7-10875H CPU @ 2.30 GHz, 16 GB RAM, 1T hard drive, and 6 GB NVIDIA RTX 2060 GPU. The algorithm adopted CUDA and CUDNN library. Python version was 3.7. Pytorch version was 1.7. All programming was conducted in Jupyter Notebook.

### 2.2. Model Establishment

CNN could realize automatic feature extraction and selection by sharing depth and weight value between network nodes. This would also help to mitigate overfitting. As CNN learning involves millions of parameters, the training process requires an enormous amount of training data and calculation. To amend such limitations, a specific strategy is introduced, which is called transfer learning [25]. The transfer learning method refers to using pre-trained deep network as a feature extractor, and fine-tuning pre-trained deep network weight with a new dataset. CNN involves different kinds of dominant pre-training structures. These structures successfully completed the classification task [26] with 1000 different categories on ImageNet dataset. Due to a strong contrast between images from the dataset and pepper images, features extracted by such a network may not be suitable for the classification of pepper samples of differing quality. To overcome this disadvantage, the fine-tuned network model and transfer learning with fine-tuned parameters were adopted for the classification of pepper using images with differing quality.

The depth of CNN had a major influence on classification precision and detection performance. Inaccuracy in classification would decrease as CNN depth increases. As the second place for ILSVRC-2014, VGGNet uses increasing depth of CNN to strengthen performance. VGGNet is obviously better than frameworks that obtained the best output in ILSVRC-2012 (AlexNet) and ILSVRC-2013 (ZFNet). Though the classification capability of VGGNet is a bit weaker than GoogLeNet (champion of ILSVRC-2014), its network structure is less complex than GoogLeNet. VGGNet is one of the most popular choices for deep learning and computer visual tasks [27].

The major philosophy of VGGNet is to adopt a 3 × 3 small size convolutional kernel and 2 × 2 max pooling filter, which enables performance promotion by depth increase, as well as a reduction in the parameter and calculation amount by decreasing the convolutional kernel size. Comparing with other deep convolutional neural networks, the structure of the VGGNet has a better processing capability for training datasets with small structure and data amount. It is also easier to be realized and has a better identification rate.

The architecture of VGG 16 includes 13 convolutional layers, 3 fully connected layers, and 5 pool layers. They are organized into 5 convolutional blocks. Each VGG convolutional block consists of one group of convolutional layer, one activation function, and one max pooling function. The blocks were configured by sequence, so as to allow for defining the output of each block as the input to the next block. With image input, VGG 16 uses feature extraction part to conduct feature extraction on the input image. Good features could make it easier to identify the target. Therefore, the feature extraction part consists of 5 convolutional blocks. Convolution is good at feature extraction. The classification part would make a classification according to features captured by the feature extraction part. The classification part usually consists of fully connected layers. Features collected by the feature extraction part are usually one-dimensional vectors, which could be directly applied to fully connected classification.

**Figure 2:**
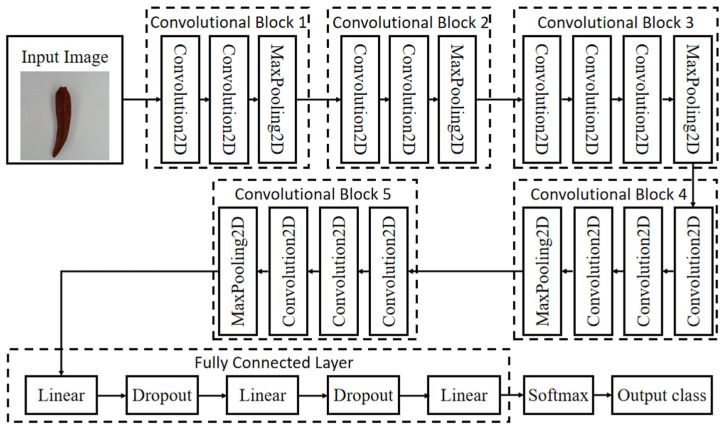
VGG 16 network structure.

In this research, the network structure of VGG 16 is shown in Figure 2. The input image size was 224 × 224 × 3. After 2 times of 3 × 3 convolutional network in Convolutional Block 1, the feature layer output by feature extraction was 64; the output was, thus, 224 × 224 × 64; then, after 2 × 2 max pooling, the output was 112 × 112 × 64. Feature layer output by 2 times of 3 × 3 convolutional network in Convolutional Block 2 was 128, and thus, the output was 112 × 112 × 128; after 2 × 2 max pooling, the output was 56 × 56 × 128. Feature layer output by 3 times of 3 × 3 convolutional network in Convolutional Block 3 was 256, and the output was 56 × 56 × 256; output after 2 × 2 max pooling was 28 × 28 × 256. Feature layer output by 3 times of 3 × 3 convolutional network in Convolutional Block 4 was 512, and thus, the output was 28 × 28 × 512; after 2 × 2 max pooling, the output was 14 × 14 × 512. The feature layer output by 3 times of 3 × 3 convolutional network in Convolutional Block 5 was 512, and thus, the output was 14 × 14 × 512; after 2 × 2 max pooling, the output was 7 × 7 × 512. The outputs were flattened by the classification part into one-dimensional vectors at the length of 25,088. Data were classified with 3 fully connected layers, respectively, with 4096, 4096, and 1000 channels. The last layer was SoftMax layer. Based on classification scores, images were classified. Scores were transferred into rates that the image was classified to different classes. The final output was in anticipation for each class.

Regarding pepper external quality classification models, VGG 16 network was required to change the output feature of the last layer from 1000 to 2. The dropout layer was added behind after each linear for regularization. Using flattened, output data of the second dropout layer, they were transferred from two-dimension tensors to one-dimensional tensors. At last, the output was put through a fully connected layer consisting of one or two neurons. Each neuron corresponds to a rate of two kinds of data. The anticipation result for Class 2 was output with SoftMax.

## 3. Results and Analysis

The experiment conducted training towards the developed VGG 16 network. The dataset was randomly divided into a training set (3260 images) and anticipation set (1396 images) with the ratio of 7:3. For the sake of better network training performance, the experiment used GPU to conduct acceleration training on training and anticipation models. Considering hardware performance and sufficient learning by the model about features in data, the research configured each test to run 100 epochs [28]. To maintain the balance between memory efficiency and memory capacity, the batch size for each training session was configured as 16.

### 3.1. Model Parameter Trimming

The learning rate is an important hyper-parameter for deep learning. It decides whether and when the target function could be converged to the local minimum. An appropriate learning rate could enable the convergence of the target function to its local minimum at appropriate timing. Therefore, under the condition of guaranteeing regular training, it could reduce the training time cost by configuring the learning rate to an appropriate range [29].

Dropout is a regularization method that could mitigate the risk of overfitting during the training process. Throughout the entire training process, some neurons were randomly removed. The rest of the neurons were involved in network training, with their weight updated [30]. This process could also help neurons learn more features, which could control overfitting.

Considering the complexity of the experiment and referring to the research results of others [31,32,33,34], the combination of dropout and learning rate trimming was adopted for model selection in the neural network development process. To realize better regularization performance, three groups of dropout value were configured (0.3, 0.5, and 0.7). To realize the best learning rate, six groups of fixed learning rate were configured (0.01, 0.001, 0.0001, 0.00001, 0.000001, and 0.0000001). In total, 18 combinations were configured for the experiment.

#### 3.1.1. Influence of Dropout on Model Performance

All groups of training precision rate, anticipation rate, training loss value, and anticipation loss values are shown in Table 1. According to the training results in Table 1, learning rate and dropout combination were of a relatively significant influence on accuracy and loss value of training and anticipation. In the training set, the learning rate was set as 0.0001, 0.00001, and 0.000001. The training precision rate was 100% at both 0.3 and 0.5 dropout configurations. When the learning rate was configured as 0.00001 and the dropout as 0.7, the training precision rate was 100%, and the training loss value was basically close to 0.

As for anticipation set, the dropout was configured as 0.3. The accuracy rate gradually increased and the loss value gradually decreased when the learning rate was configured between 0.01 and 0.000001. Comparing with the 0.000001 learning rate, the 0.0000001 learning rate was of a smaller accuracy rate and larger loss value. When the dropout was configured as 0.3, the best learning rate was 0.000001, the anticipation precision rate was 98.14%, and the anticipation loss value was 0.0984. When the dropout was configured as 0.5 and 0.7, the accuracy rate gradually increased, and the loss value gradually decreased when the learning rate was configured between 0.01 and 0.00001. The accuracy rate gradually decreased, and the loss value gradually increased when the learning rate was configured between 0.00001 and 0.00000001. When the dropout was configured as 0.5, the best learning rate was 0.00001, the anticipation precision rate was 98.07%, and the anticipation loss value was 0.0669. When the dropout was configured as 0.7, the best learning rate was 0.00001, the anticipation precision rate was 97.99%, and the anticipation loss value was 0.1193. Comparing with the combination with the same learning rate but with the dropout configured as 0.5 and 0.7, the training and anticipation precision rates were both lower than the combination with the dropout configured as 0.3. This proved that configuring the dropout as 0.3 enabled the best generalization ability, which could avoid overfitting of the model.

#### 3.1.2. Influence of Learning Rate on the Model

**Figure 3:**
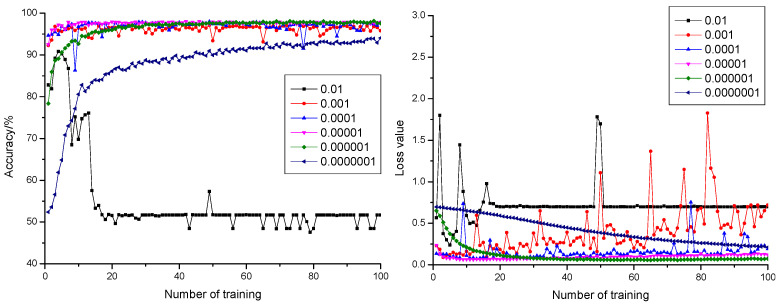
The anticipation precision rate curve and anticipation loss value curve under a different learning rate when dropout = 0.3.

**Figure 4:**
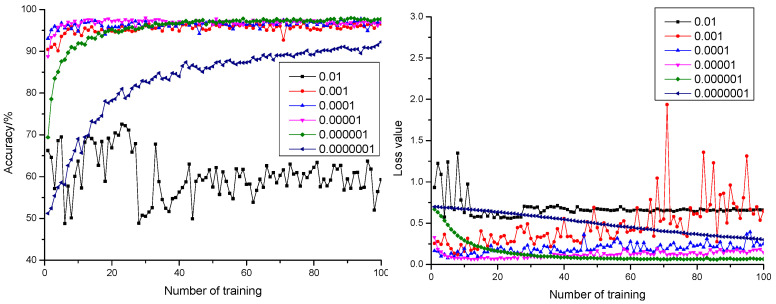
The anticipation precision rate curve and anticipation loss value curve under a different learning rate when dropout = 0.5.

**Figure 5:**
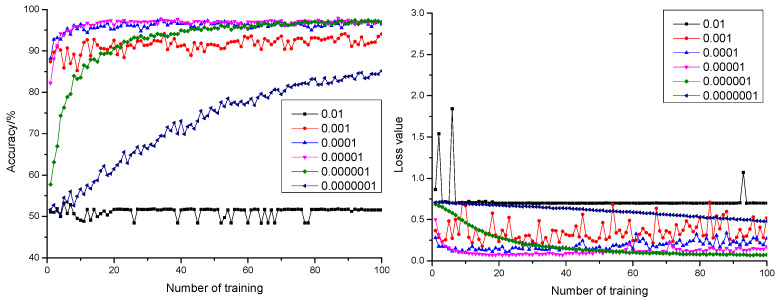
The anticipation precision rate curve and anticipation loss value curve under a different learning rate when dropout = 0.7.

Curves of anticipation precision rate and anticipation loss value when the dropout was configured as 0.3, 0.5, and 0.7 are shown in Figure 3, Figure 4 and Figure 5, respectively. According to Figure 3, Figure 4 and Figure 5, it could be learnt by comparing with the precision rate and anticipation loss value under five learning rates from 0.001 to 0.0000001 that the larger the learning rate was, the faster the convergence rate would be. The anticipation precision rate and anticipation loss value appeared to fluctuate relatively in early stage of the training model when the learning rate was configured as 0.01 and became stable after training 20 times. The precision rate was about 55%, and the loss value was approximately 0.7. The anticipation precision rate curves were choppy and had low accuracy; the anticipation loss value curves were basically stable and converged, but the loss value is high, which is not suitable for normal training. When the learning rate was configured as 0.001, the convergence rate of the anticipation precision rate was the fastest. Certain training showed fluctuation and relatively larger fluctuation of the loss value, which required optimization towards model parameters. When the learning rate was configured as 0.0001, the convergence rate of the anticipation precision rate was relatively fast. The precision rate curve also converged stably. Comparing with the learning rate configured as 0.001, the fluctuation was relatively smaller. This indicated that the model was of insufficient fitting capability and requires further optimization towards the model parameters. When the learning rate was configured as 0.00001, the convergence rate of the anticipation precision rate was relatively fast. The precision rate curve converged stably. The anticipation loss value curve decreased at first and then increased, which indicated that the model was at its local optimum. When the learning rate was configured as 0.000001, the convergence rate of the anticipation precision rate was relatively slow. The precision rate curve converged stably. The anticipation loss value curve converged stably and resulted in a minimum loss value, which proved the strong fitting capability of the model. When the learning rate was configured as 0.0000001, the convergence rate of the anticipation precision rate was the slowest. Both the precision rate curve and the anticipation loss value curve did not completely converge, which proved that the learning rate configuration was small, and the convergence of the model was slow. The time cost for locating an optimal value needed to be increased.

Integrating with Table 1, comparing with 18 combinations, the highest anticipation precision rate 98.14% and the minimum anticipation loss value 0.06 were realized with the learning rate configured as 0.000001 and dropout as 0.3. This was the best performance, comparing with other combination models.

#### 3.1.3. Influence of Batch Normalization Layer on Model Performance

**Figure 6:**
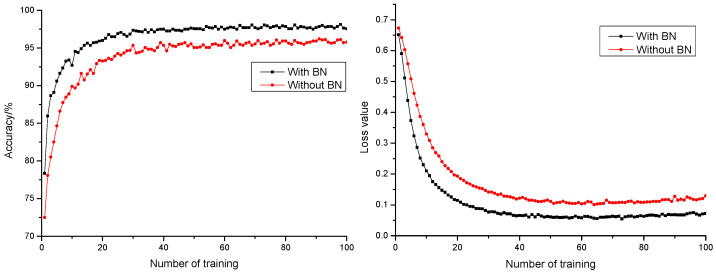
Anticipation precision rate and anticipation loss value curve with or without normalization.

Batch normalization (BN) layer refers to conducting a normalization process to output layer. The output would be subjected to normal distribution with an average of 0 and variance of 1, which could avoid the issue of internal covariate shift [35]. During trainings, input to each layer was normalized by calculating the average value and variance for the data of the former layer. With the normalization process towards input to the input layer, change to internal neuron distribution was reduced. A difference in ranges between different samples was also reduced. Most gained data distributed in unsaturated regions. This guaranteed efficient feedback of the gradient, which avoided gradient vanishing and explosion, accelerated network convergence rate, and optimized network structure. Anticipation precision rate and anticipation loss value curve of models with or without a BN layer are shown in Figure 6. It could be found that the addition of a BN layer to models could effectively accelerate network convergence rate, promote anticipation precision rate of the model by 1.94%, and reduce model anticipation loss value by 0.0482. This resulted in a better and more stable anticipation performance.

#### 3.1.4. Influence of Activation Function on Model Performance

**Figure 7:**
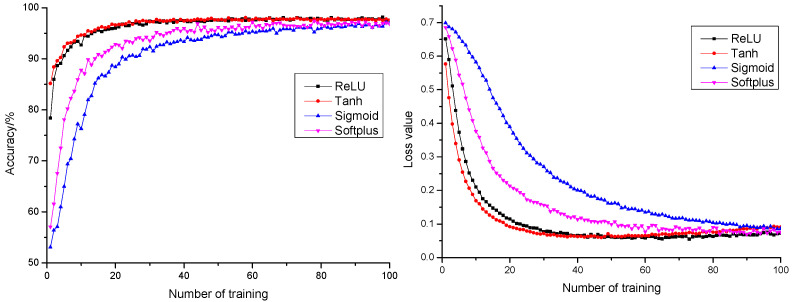
The anticipation precision rate and anticipation loss value curve of different activation functions.

Activation function is of critical function to neural network models in learning and comprehending complicated and non-linear functions [36]. Data distribution is usually non-linear, yet calculation of a network model is usually linear. Introduction of activation function means the introduction of non-linearity into a neural network to strengthen learning capability of the network. To improve the precision rate and stability of the pepper classification model, this research conducted performance influence and comparative analysis on the model with four activation functions: ReLU, Tanh, Sigmoid, and Softplus. The anticipation precision rate and anticipation loss value curves of different activation functions are shown in Figure 7. All four activation functions resulted in a final anticipation precision rate higher than 97% and an anticipation loss value less than 0.1. It could be learnt from the figure that ReLU and Tanh, compared with the anticipation precision rate curve and anticipated loss value curve of the Sigmoid and Softplus model, had faster convergence rates, which saved time cost of optimization and proved strong fitting capability for the model. The anticipation loss value curve of ReLU and Tanh both decreased first and then increased. Compared with the Tanh function, the increase amplitude of the ReLU function was small. After epoch 50 times, the maximum difference in anticipation loss by ReLU function was less than 0.02, which tended to be basically stable. Therefore, ReLU was selected as the activation function in this study.

### 3.2. Model Performance Comparison

**Figure 8:**
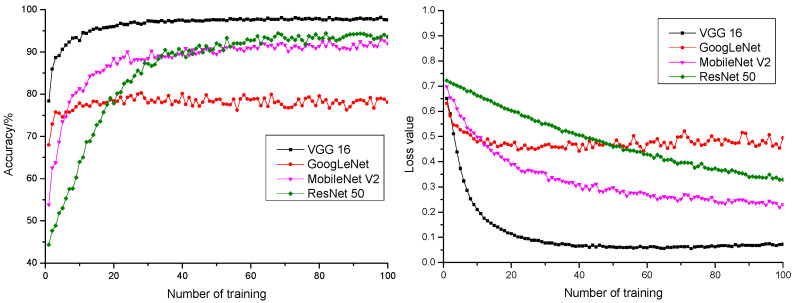
The anticipation precision rate and anticipation loss value curve of different activation functions.

To verify the effectiveness of the model in classifying normal and inferior pepper, this research, after parameter optimization, applied VGG 16 and other deep learning classification models (ResNet 50, MobileNet V2, and GoogLeNet) in pepper classification. The training precision rate, anticipation precision rate, training loss value, and anticipation loss value of all model experiments are shown in Table 2. The anticipation precision rate and anticipation loss value curve of different models are shown in Figure 8.

It could be learnt from Figure 8 that the anticipation precision rate curve and anticipation loss value curve of VGG 16 model were of the fastest convergence rate and stable convergence, the highest anticipation precision rate, and the lowest anticipation loss value. It could be learnt from the comparison that the VGG 16 model was more suitable for pepper quality detection.

## 4. Discussion

In this experiment, transfer learning combined with convolutional neural network was used to detect external quality of pepper, and VGG 16 was used to train with different parameters, so as to obtain the optimal accuracy and loss value. In this experiment, the best results were obtained when the dropout was set to 0.3, the learning rate was set to 0.000001, a BN layer was added as the model normalization method, and ReLU was used as the activation function.

In external quality detection for pepper, Sun judged the external quality of pepper with its color feature [37] and conducted classification based on the relationship between external quality feature and color component. This resulted in an 96% precision rate. Cui designed a Hough forest classifier based on machine-vision-based pepper external quality detection technology for pepper identification and realized a 90% identification rate [38]. Compared with the abovementioned studies, this research integrated VGG 16 on the basis of transfer learning for pepper external quality detection. It realized a 98.14% anticipation precision rate, which has a brighter outlook for detection precision and cost. The classification precision of this research has no significant difference with former research on machine-learning-based pepper external quality detection [39].

In the VGGNet network applied in agricultural product quality detection, Nasiri used VGG 16 to classify jujube fruits at different maturity stages and achieved an overall classification accuracy of 96.98%. Experimental results on this model show that this model is superior to the traditional feature engineering-based jujube fruit image classification method [40]. The papaya fruit classification method proposed by Behera based on transfer learning combined with the VGG 19 method achieves 100% accuracy, which is 6% higher than the machine learning method and requires less training time. Based on the above studies, it is feasible to apply VGG 16 to the external quality detection of pepper [41].

Traditional machine learning requires manual image feature information extraction, which is restricted to data processing capability and segmentation requirements. Compared with machine learning technology, CNN requires no process of feature extraction and feature selection. Though CNN integration by transfer learning requires a complicated system framework, long training duration, and large dataset, it could guarantee model performance while promoting the training speed and generalization ability of the model. Considering the future development of the pepper industry, integrating transfer learning with CNN could be a solution for automatic pepper external quality classification that could promote detection efficiency and reduce production cost. To further consummate online pepper quality models, future studies could accelerate model training speed and reduce model size while maintaining the stability and accuracy of the model, which would be of important production meaning and economic benefit to the pepper industry.

## 5. Conclusions

This research integrated transfer learning method with VGG 16 model and realized model optimization by fine tuning a combination of dropout and learning rate in the model. It explored the influence of adding a BN layer on model performance, compared the influence of different activation functions on model performance, and established a pepper selection model. It also conducted comparison analysis with ResNet 50, MobileNet V2, and GoogLeNet models in transfer learning after parameter optimization and obtained conclusions as follows:

1. According to the comparison of 18 dropout and learning rate combinations, appropriate configuration of dropout and learning rate under the condition of maintaining stable training could promote generalization performance and training precision of the model. Therefore, the configuration of dropout as 0.3 and learning rate as 0.000001 could realize the highest anticipation precision rate of 98.14% and the smallest anticipation loss value of 0.0669.
2. With the addition of a BN layer as a method for model normalization, the model could effectively accelerate network convergence rate, promote the model anticipation precision rate by 1.94%, and reduce the model anticipation loss value by 0.0482. It promoted generalization capability of the model and realized a better and more stable model anticipation performance. With ReLU as activation function, the model had a faster convergence rate. This saved time cost for optimization, which indicated the strong fitting capability of the model.
3. A comparison was also conducted with ResNet 50, MobileNet V2, and GoogLeNet models in transfer learning after parameter optimization. It turned out that the VGG 16 model gave the best performance with 100% training precision rate, 98.14% anticipation precision rate, the fastest convergence rate, and stable convergence in anticipation precision rate curve and anticipation loss value curve, as well as the highest anticipation precision rate and the lowest anticipation loss value. Therefore, VGG 16 was more suitable for pepper quality detection.

In this study, we only consider to improve the accuracy of the model by trimming the model parameters, and there are some limitations in our work. In future research, we will use more precise descriptions to study the relationship between parameters and propose new models or optimization techniques to improve model performance.

## Figures and Tables

**Table 1:** Precision rate and loss value of training and anticipation in the model.

Experiment Code	Learning Rate	Dropout	Precision Rate in Training	Anticipation Precision Rate	Loss Value in Training	Anticipation Loss Value
1	0.01	0.3	90.64%	90.83%	0.293	0.2997
2		0.5	70.92%	72.56%	0.5714	0.5616
3		0.7	50.28%	53.22%	0.8628	0.7067
4	0.001	0.3	99.97%	97.35%	0.0017	0.1388
5		0.5	99.48%	96.78%	0.0279	0.3399
6		0.7	95.92%	94.13%	0.1659	0.1731
7	0.0001	0.3	100.00%	97.99%	0.0001	0.1152
8		0.5	100.00%	97.78%	0.0001	0.1723
9		0.7	99.91%	97.64%	0.0049	0.1331
10	0.00001	0.3	100.00%	98.07%	0.0001	0.0808
11		0.5	100.00%	98.07%	0.0001	0.0984
12		0.7	100.00%	97.99%	0.0002	0.1193
13	0.000001	0.3	100.00%	98.14%	0.00001	0.0669
14		0.5	100.00%	97.99%	0.0012	0.0647
15		0.7	99.88%	97.42%	0.0172	0.0732
16	0.0000001	0.3	95.28%	94.05%	0.1933	0.2106
17		0.5	92.82%	92.26%	0.2844	0.2996
18		0.7	85.17%	86.35%	0.4779	0.4655

**Table 2:** Precision rate and loss value of training and experiment in different models.

Model	Precision Rate in Training	Anticipation Precision Rate	Loss Value in Training	Anticipation Loss Value
VGG 16	100.00%	98.14%	0.0001	0.0669
GoogLeNet	98.13%	80.30%	0.0692	0.4541
MobileNet V2	92.82%	92.91%	0.2258	0.2357
ResNet 50	96.47%	94.41%	0.3079	0.4001
